# E41K mutation activates Bruton’s tyrosine kinase by stabilizing an inositol hexakisphosphate-dependent invisible dimer

**DOI:** 10.1016/j.jbc.2024.107535

**Published:** 2024-07-04

**Authors:** Subhankar Chowdhury, Manas Pratim Chakraborty, Swarnendu Roy, Bipra Prasad Dey, Kaustav Gangopadhyay, Rahul Das

**Affiliations:** 1 Department of Biological Sciences, Indian Institute of Science Education and Research Kolkata, Mohanpur, India; 2 Centre for Advanced Functional Materials, Indian Institute of Science Education and Research Kolkata, Mohanpur, India

**Keywords:** kinase, cell signaling, BTK, inositol hexakisphosphate, B-cell receptor

## Abstract

Bruton’s tyrosine kinase (BTK) regulates diverse cellular signaling of the innate and adaptive immune system in response to microbial pathogens. Downregulation or constitutive activation of BTK is reported in patients with autoimmune diseases or various B-cell leukemias. BTK is a multidomain protein tyrosine kinase that adopts an Src-like autoinhibited conformation maintained by the interaction between the kinase and PH-TH domains. The PH-TH domain plays a central role in regulating BTK function. BTK is activated by binding to PIP_3_ at the plasma membrane upon stimulation by the B-cell receptor (BCR). The PIP_3_ binding allows dimerization of the PH-TH domain and subsequent transphosphorylation of the activation loop. Alternatively, a recent study shows that the multivalent T-cell-independent (TI) antigen induces BCR response by activating BTK independent of PIP_3_ binding. It was proposed that a transiently stable IP_6_-dependent PH-TH dimer may activate BTK during BCR activation by the TI antigens. However, no IP_6_-dependent PH-TH dimer has been identified yet. Here, we investigated a constitutively active PH-TH mutant (E41K) to determine if the elusive IP_6_-dependent PH-TH dimer exists. We showed that the constitutively active E41K mutation activates BTK by stabilizing the IP_6_-dependent PH-TH dimer. We observed that a downregulating mutation in the PH-TH domain (R28H) linked to X-linked agammaglobulinemia impairs BTK activation at the membrane and in the cytosol by preventing PH-TH dimerization. We conclude that the IP_6_ dynamically remodels the BTK active fraction between the membrane and the cytoplasm. Stimulating with IP_6_ increases the cytosolic fraction of the activated BTK.

Bruton’s tyrosine kinase (BTK) is an indispensable signaling module of innate and adaptive immune response against pathogens. BTK downregulation in patients with X-linked agammaglobulinemia (XLA) causes recurring bacterial and viral infections (1, 2, 3, 4). On the other hand, constitutive activation of BTK is often linked to several forms of B-cell leukemias and lymphomas like chronic lymphocytic leukemia (5), and diffuse large B cell lymphoma (ABC-DLBCL) (6), suggesting a critical regulatory function of BTK in B-cell development and proliferation (7). Thus, BTK has been an important pharmacological target for the treatment of various B-cell malignancies (8, 9, 10).

In macrophages, a component of innate immunity, BTK phosphorylates the Toll-like receptors to initiate an antiviral response (11). In B-cells, a component of adaptive immunity, BTK is essential for connecting the B-cell antigen receptor (BCR) activation to the downstream calcium flux (12, 13, 14). BTK is a Tec family kinase (15) that shares a homologous structural architecture with the Src kinases, where the kinase domain is flanked by two N-terminal Src homology domains, SH3 and SH2 (Fig. 1*A*) (16, 17, 18). BTK has an additional lipid binding pleckstrin homology (PH) domain fused to the Tec homology (TH) domain at the N-terminal of the SH3 domain (19, 20). Unlike Src kinase, where the autoinhibited conformation is stabilized by the C-terminal phosphotyrosine residue (16, 17, 18), in BTK, the PH-TH domain functions as the negative regulator (21, 22, 23). In the autoinhibited state, the SH3 and SH2 domains assemble at the back of the kinase domain (Fig. 1*B*) (16, 17, 18, 22). The interaction of the PH-TH domain and the kinase domain maintains the autoinhibited structure, blocking the activation loop face of the kinase domain, which occludes the lipid binding to the PH-TH domain (22, 24, 25).

**Figure 1:**
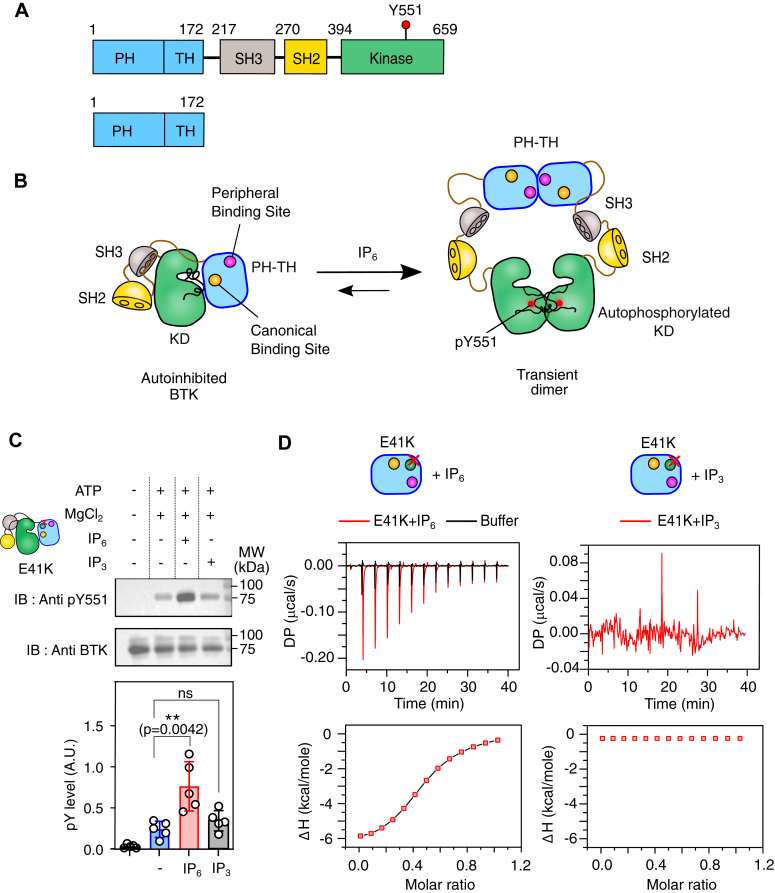
**Activation of BTK E41K mutant by Inositol Hexakisphosphate (IP**_**6**_**).***A*, schematic representation of domain architecture of Bruton’s Tyrosine Kinase and PH-TH domain used in this study. *B*, mechanism of Inositol Hexakisphosphate (IP_6_) dependent activation of Bruton’s Tyrosine Kinase (22). *C*, *top panel*: representative immunoblot of Y551 autophosphorylation in the full-length BTK E41K (2 μM) mutant with 100 μM IP_6_ or IP_3._*Bottom panel*: densitometric analysis of the immunoblots of BTK E41K mutant activated with IP_6_ or IP_3_. The error bar represents standard deviations from five independent experiments. Data are presented as mean values ± SD from five independent experiments. Data analyses were performed using GraphPad Prism version 9.5.1. An unpaired two-tailed *t* test was used to calculate significance. *D*, *top panel*: Isothermal Titration Calorimetric (ITC) measurement of IP_6_ (left) or IP_3_ (right) binding to E41K mutant of an isolated PH-TH domain. For each titration, 25 μM of PH-TH^E41K^ was titrated with 137 μM of ligand. Bottom panel: solid line represents the two-site independent binding fitting of the ITC titration of IP_6_ and indicated PH-TH domain. The experimental points are indicated in red square. The ITC titration of IP_3_ and PH-TH^E41K^ domain could not be fit to any model. The plots were generated using Origin Pro 2020b.

Antigen binding to BCR activates BTK by recruiting kinase to the plasma membrane (Fig. S1*A*) (26, 27). The localization of the BTK to the membrane is mediated by the binding between the phosphatidylinositol (3,4,5)-triphosphate (PIP_3_) and the canonical lipid-binding site of the PH-TH domain (Fig. S1*A*) (4, 22, 28, 29). Binding to PIP_3_ removes the inhibitory interaction, allowing BTK to dimerize (28, 30) and rapidly phosphorylate the tyrosine residue in the activation loop (13, 31, 32). The PIP_3_ induces dimerization of the PH-TH domain (called Saraste dimer) where hydrophobic dimer interfaced (called Saraste interface) is constituted by α1-helix, β1 and β3-β4 loop (22, 28, 29). At the membrane, the PH-TH dimer is stabilized by a second PIP_3_ binding to the peripheral lipid binding site (Fig. S1*A*), which imparts a switch-like property to the kinase (30).

Alternatively, recent studies showed that inositol hexakisphosphate (IP_6_) is required for a T-cell-independent BCR response against bacterial and viral infection (33). Deficiency of inositol polyphosphate multikinase in mice, the enzyme that generates the precursor for IP_6_ biosynthesis from inositol tetrakisphosphate (IP_4_) (34), impairs BTK activity and attenuates calcium signaling in B-cells. The IP_6_ was previously shown to induce BTK activation in solution by the same PH-TH Saraste dimer, where two molecules of IP_6_ bind to the canonical site and peripheral sites, respectively (Fig. 1*B*) (22), suggesting IP_6_ activates BTK independently of its membrane localization. Mutations that impair IP_6_ binding to the peripheral sites inhibit BTK activation in the solution. However, no PH-TH dimer in solution has yet been determined. It was speculated that the PH-TH domain might form a transiently stable dimer in the presence of IP_6_ (22). Therefore, the lack of evidence for an IP_6_-dependent BTK dimer makes the functional relevance of T-cell-independent BCR response to pathogens uncertain.

In the present study, we focused on an E41K mutant of the PH-TH domain that activates BTK constitutively (35) and binds to IP_6_ with a higher affinity than the wild-type (4). We observed that, unlike wild-type BTK (22), the E41K mutant is activated by IP_6_ in solution by stabilizing a PH-TH dimer, which was otherwise invisible by most biophysical techniques due to the transient nature of the dimer. Our mutation studies suggest that the PH-TH dimerization is mediated by an alternate dimer interface, where an IP_6_ molecule holds two lateral PH-TH domains together. The R28H mutation found in the canonical lipid-binding site of the PH-TH domain in patients with XLA (4, 36) prevents IP_6_-dependent PH-TH dimerization and inhibits BTK activation. In cells, higher IP_6_ concentration increases the cytosolic fraction of activated BTK. Finally, we presented a mechanism explaining how IP_6_ remodels the equilibrium, like a switch, between the membrane fraction and cytosolic fraction of BTK in the cell.

## Results and discussion

### IP_6_ activates BTK E41K mutant in solution

**Figure 2:**
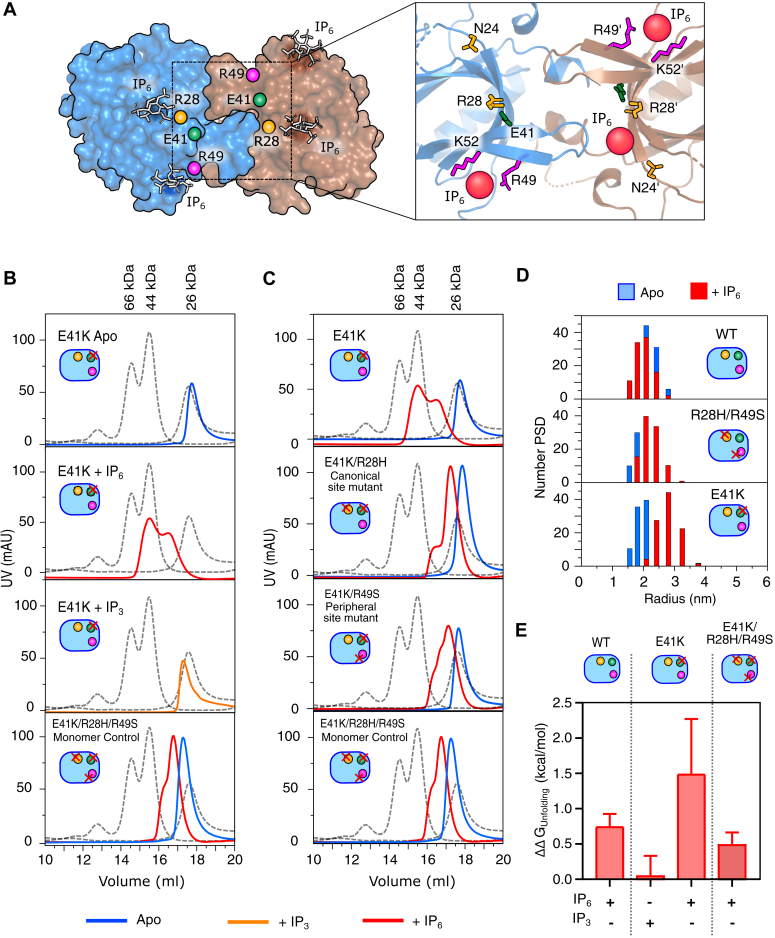
**Probing the IP**_**6**_**-dependent PH-TH dimer in solution.***A*, the space-filled model of BTK PH-TH Saraste dimer in complex with IP_6_ (PDB 4Y94). The canonical and peripheral ligand binding site is shown in the inset. *B* and *C*, representative elution profiles of indicated constructs of PH-TH domain in the presence (300 μM) or absence of IP_6_ or IP_3_ when passed through a size-exclusion column. The triple mutant of the PH-TH domain (E41K/R28H/R49S) is used as monomer control. The dotted line represents the elution profiles of standard protein mixtures containing BSA (66 kDa), Ovalbumin (44 kDa), and ULP1 (26 kDa). The plots were generated by XMGRACE Ver 5.1.25. *D*, dynamic light scattering measurement of indicated constructs of PH-TH domain in the presence (*red bar*) or absence (*blue bar*) of IP_6_. The plots were generated using Origin Pro 2020b. *E*, ΔΔG_unfolding_ of ligand-bound BTK PH-TH constructs derived from the thermal denaturation profile measured using a Circular Dichroism (CD) spectrophotometer (Fig. S2*D*). ΔΔG_unfolding_ was derived at the T_m_ of the respective *apo* state of indicated constructs. Data are presented as mean values ± SD from three independent experiments. The plots were generated using GraphPad Prism version 9.5.1. See Table S2 and Fig. S2.

The E41K mutation is located at the β3-β4 loop of the PH-TH domain (Fig. 2*A*). The crystal structure of an isolated PH-TH^E41K^ mutant was solved in a complex with D-myo-inositol 1,3,4,5-tetra-kisphosphate (IP_4_) (37). The lysine residue (at 41 position) does not directly contact the IP_4_ molecule in the structure at the canonical binding site (37). Instead, the lysine creates an overall positively charged environment, allowing the second IP_4_ molecule to bind, leading to spontaneous membrane recruitment of the BTK, allowing autophosphorylation of Y551 at the activation loop, and enhanced Ca^2+^ signaling (35, 37, 38). The high structural homology (RMSD of 0.43 Å) between the PH-TH^E41K^ mutant in complex with IP_4_ (PDB ID: 1BWN) (37) and the wild-type PH-TH domain in complex with IP_6_ (PDB ID: 4Y94) (22) does not explain the observed functional difference (Fig. S1*B*). However, a higher binding affinity of the E41K mutant for IP_6_ (4) suggests that the mutation may stabilize a PH-TH dimer. To find out if IP_6_ also activates BTK carrying an E41K mutation in solution, we purified the full-length BTK (denoted as BTK^E41K^) and an isolated PH-TH domain with E41K mutant (denoted as PH-TH^E41K^) and determined the phosphorylation of Y551 and binding of IP_6_, respectively (Fig. 1, *C* and *D*). Without any ligand, we observed that the BTK^E41K^ spontaneously phosphorylated at Y551 (Fig. 1*C*). On the other hand, adding IP_6_ significantly increases the Y551 phosphorylation compared to IP_3_, the unliganded state, and the wild-type BTK (Fig. S1, *C* and *D*). As reported previously, IP_3_ does not induce phosphorylation of Y551 because the PH-TH^E41K^ domain does not bind to IP_3_ (Fig. 1, *C* and *D* and Table S1) (4). On the other hand, PH-TH^E41K^ binds to IP_6_ with two distinct dissociation constants (*K*_*d1*_ = 152 ± 18 nM and *K*_*d2*_ = 1.3 ± 0.4 μM) when the ITC data was fitted to a two-site independent binding model (Fig. 1*D* and Table S1). We observed that the *K*_*d1*_ and *K*_*d2*_ represent the strong and weak IP_6_ binding sites, respectively, which may correspond to the canonical and peripheral lipid binding sites in the PH-TH domain, respectively (22). We asked if the improved activation of BTK is due to the greater stability of the IP_6_-dependent dimer in the solution.

### IP_6_ promotes PH-TH dimerization

In the crystal structure of the PH-TH domain in complex with IP_6_, the Saraste-dimer interface is comprised of antiparallel interaction between the adjacent β3-β4 loop and α1-helix of each PH-TH domain (Fig. 2*A*) (22). However, due to the transient nature of the hydrophobic interaction at the Saraste-dimer interface, no IP_6_-dependent PH-TH dimer has yet been detected in the solution. Since PH-TH^E41K^ binds IP_6_ with a stronger affinity compared to the wild-type (4), we speculate if the activation of BTK^E41K^ is due to forming a stable IP_6_-dependent PH-TH dimer. We probed the PH-TH^E41K^ dimer from the mobility of the protein by size-exclusion chromatography and from dynamic light scattering (DLS) measurements (Fig. 2). In our assays, we used double mutants (R28H and R49S at the canonical and peripheral sites, respectively) of the PH-TH domain made in the wild-type or E41K background as a monomer control. The anticipated molecular weight and theoretical hydrodynamic radius of the PH-TH dimer are 38 kDa and 3.0 nm, respectively (Fig. S2*B*). As reported, we could not detect IP_6_-dependent dimers for the wild-type PH-TH domain by size-exclusion chromatography and DLS experiment (Figs. 2*D* and S2, *A*–*C*). We observed a minor shift in the retention time for the IP_6_ bound PH-TH domain compared to the *apo*-state, and the hydrodynamic radius (2.18 ± 0.10 nm) was close to a monomer (Figs. 2*D* and S2, *A*–*C*).

On the contrary, the PH-TH^E41K^ in the presence of IP_6_ migrated close to a dimer (44 kDa) species in a size-exclusion column (Fig. 2*B*). The hydrodynamic radius of 3.14 ± 0.48 nm suggests that the PH-TH^E41K^ in the presence of IP_6_ forms a stable homodimer (Figs. 2*D* and S2, *B* and *C*). We noted that PH-TH^E41K^ does not dimerize in the *apo*-state or in the presence of IP_3_ (Fig. 2*B*). To find out if IP_6_ induces structural stability in the PH-TH domain, we compare Gibbs free energy for unfolding (ΔG_unfolding_) of the PH-TH constructs derived at the melting temperature (T_m_) of the respective *apo* state (Table S2) (39, 40). The T_m_ was determined from the thermal denaturation profile of the PH-TH domains recorded in the presence and absence of ligands (Fig. S2*D*) (39, 41). Comparison of change in ΔG_unfolding_ of the ligand-bound PH-TH constructs with respect to the corresponding apo state (ΔΔG_unfolding_ = ΔG_*holo*_ – ΔG_*apo*_), we observed that the PH-TH^E41K^ in complex with IP_6_ (1.49 ± 0.79 kcal/mole) is structurally more stable than IP_3_ complex or wild-type PH-TH domain (Figs. 2*E*, S2*D*, and Table S2). Our data suggests that the activating mutant stabilizes the PH-TH domain structure compared to inactivating mutants or ligands (Fig. 2*E*) (29).

### Activation of BTK E41K mutant is mediated by the IP_6_-dependent PH-TH dimer

The β3-β4 segment crosstalk with the canonical lipid binding site, peripheral lipid binding site, and the Saraste dimer interface. Recent studies suggest that PIP_3_ binding to the canonical and peripheral lipid binding is important for membrane docking and dimerization of BTK (29, 30). Mutation, such as R28H found in patients with XLA, at canonical lipid binding pocket impairs PIP_3_ binding and down-regulates BTK signaling (42). We next investigate whether the down-regulation of BTK in XLA mutants found at the PH-TH domain is due to impaired dimerization.

**Figure 3:**
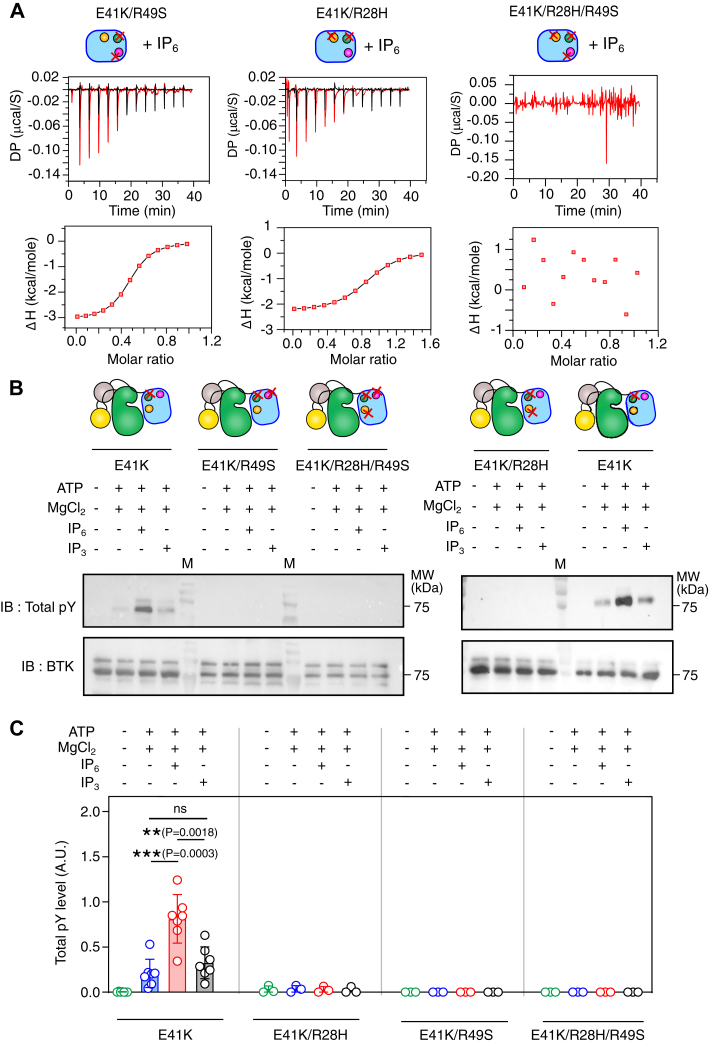
**Functional analysis of IP**_**6**_**mediated activation of BTK E41K mutant.***A*, ITC titration of IP_6_ to the indicated mutants of the PH-TH domain of BTK. For each titration, 25 μM of PH-TH^E41K^ was titrated with 137 μM of ligand. *Top panel*: *red lines* indicate protein and ligand titration, and the *black line* represents buffer-to-buffer titration. *Bottom panel*: *solid line* represents the one-site binding fitting of the ITC titration of IP_6_ and indicated PH-TH domain. The experimental points are indicated in red square. The ITC titration of IP_3_ and PH-TH^E41K^ domain could not be fit to any model. The plots were generated using Origin Pro 2020b. *B*, representative immunoblot of indicated mutants of full-length BTK with 100 μM IP_6._ The level of autophosphorylation is determined with a total anti-phosphotyrosine antibody. *C*, densitometric analysis of the immunoblots in panel B. Data are presented as mean values ± SD from 5 to 7 independent experiments for E41K and 3 independent experiments for indicated double and triple mutants of PH-TH domain (E41K/R28H, E41K/R49S, E41K/R28H/R49S). An unpaired two-tailed *t* test was used to calculate significance. Data analyses were performed using GraphPad Prism version 9.5.1.

We begin with characterizing IP_6_ binding and dimerization of the R28H mutant (at the canonical site) and R49S mutant (at the peripheral site) created in the PH-TH^E41K^ background (Figs. 2*C* and 3*A*, and Table S1). We also compared the ability to autophosphorylate full-length BTK E41K mutant (BTK^E41K^) bearing a mutation in the canonical (R28H) or peripheral (R49S) lipid binding site (Fig. 3, *B* and *C*). We observed that the R28H mutation impairs IP_6_ binding to the canonical site but retains the weak IP_6_ binding to the peripheral lipid binding site with *K*_*d*_ of 2.4 ± 0.15 μM (Fig. 3*A* and Table S1). The R49S mutation in the peripheral lipid binding site impairs IP_6_ binding at the peripheral site but retains the strong IP_6_ binding to the canonical lipid binding site with *K*_*d*_ of 622 ± 77 nM. However, in the size-exclusion chromatography, we observed that none of the mutants dimerize in the presence of IP_6_ and migrate along with the monomer control (Fig. 2*C*). As anticipated, impaired PH-TH dimerization impairs IP_6_-dependent autophosphorylation of BTK^E41K^ R28H or R49S mutants (Fig. 3, *B* and *C*). Our data shows that the XLA mutation (R28H) and mutation at the peripheral lipid binding site (R49S) impair BTK function by destabilizing the Saraste dimer interface, suggesting an allosteric crosstalk between the two lipid binding pockets and the dimer interface.

### E41K mutation promotes spontaneous localization of BTK to the plasma membrane

In B-cells, BTK is activated by localizing the kinase to the membrane following antigen binding to the BCR (26, 43). The E41K mutation spontaneously localizes the kinase to the plasma membrane when expressed in NIH 3T3 cells and *trans*-phosphorylate Y551 in the activation loop (13, 35, 38). At the membrane, the binding to PIP_3_ at the canonical and peripheral lipid binding sites activates BTK by stabilizing the Saraste dimer (30). Recently, IP_6_ was shown to regulate BTK function during BCR response to T-cell independent antigens (33). We wonder about how IP_6_ may remodel the BTK activation profile at the membrane.

**Figure 4:**
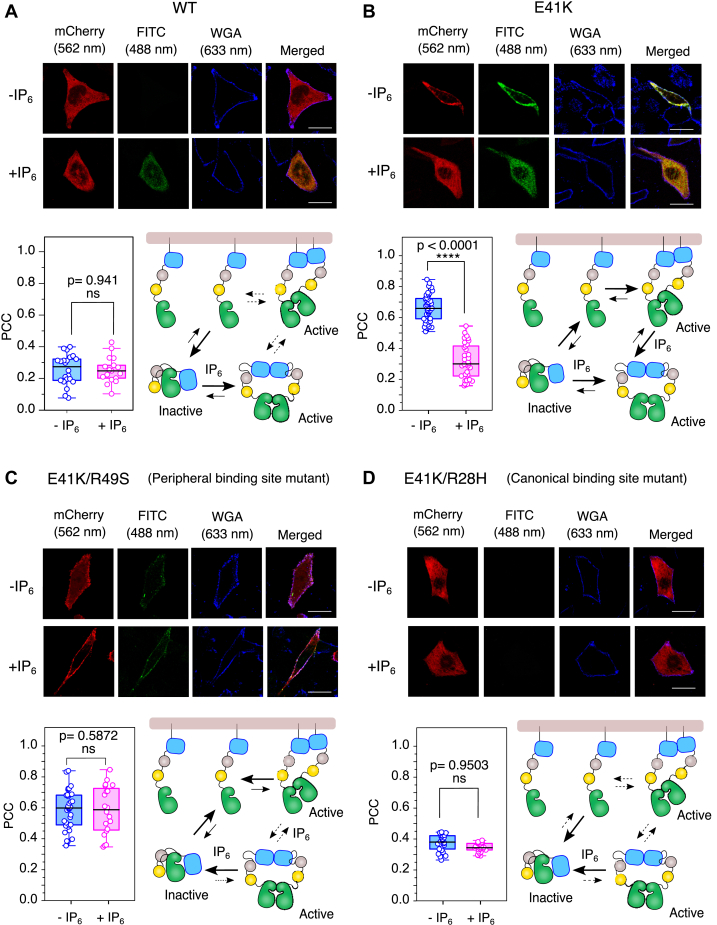
**IP**_**6**_**-dependent activation of BTK in CHO cell line.***A*–*D*, confocal images of transiently transfected BTK constructs fused to mCherry in CHO cell lines. The BTK expression level is shown in red (λ_ex_ = 552 nm, λ_em_ = 586–651 nm), and the phosphorylation status is shown in green (λ_ex_ = 488 nm, λ_em_ = 505–531 nm). The blue represents the plasma membrane stained with Wheat Germ Agglutinin (WGA) fused to Alexa 633 (λ_ex_ = 633 nm, λ_em_ = 647–692 nm). In each panel, the first column is the expression level of the indicated construct of BTK-mCherry. The second column represents the phosphorylation level Y551, determined with a specific anti-pY551 antibody followed by secondary staining with FITC conjugated secondary antibody. The third column shows the cell periphery (plasma membrane) labeled with WGA-Alexa633, and the last column is an overlay. The quantification of colocalization between BTK and WGA by Pearson's correlation coefficient (PCC) is shown in the bottom left of each panel. n = 22 to 25 over three independent experiments. scale bar = 30 μm. Boxplots represent quartiles. The data points outside the whisker range are set as outliers. The *black line* inside the box represents the median value. An unpaired two-tailed *t* test was used to calculate significance. Boxplots were generated using Origin Pro 2020b. Image analysis was done using Fiji Ver 1.54f. The bottom right in each panel shows the schematic model of BTK activation by IP_6_.

**Figure 5:**
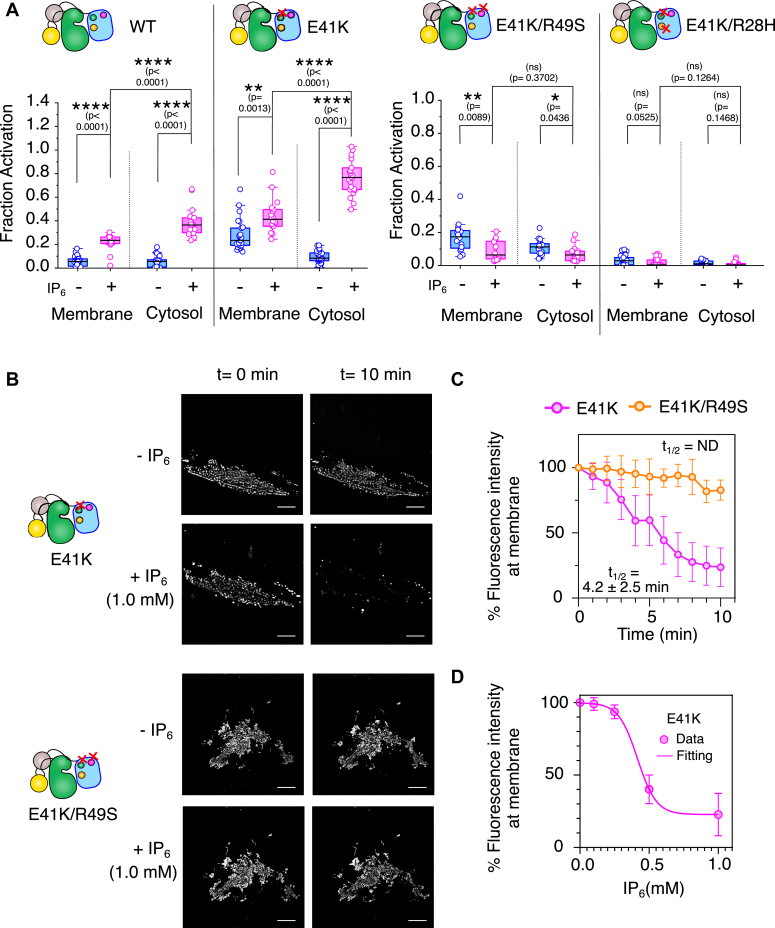
**Determination of IP**_**6**_**-dependent autophosphorylation and plasma membrane resident time of BTK.***A*, plots of fraction phosphorylated for the indicated BTK construct transiently expressed in CHO cell lines. The autophosphorylation level Y551 was determined with an anti-pY551 antibody. The phosphorylation of BTK localized at the plasma membrane and in the cytoplasm was measured in the presence and absence of IP_6_. The fraction was obtained by normalizing the pY551 level (FITC intensity) by the BTK expression level (mCherry intensity). n = 22 to 25 cells over 5 independent experiments. Boxplots represent quartiles. The data points outside the whisker range are set as outliers. The *black line* inside the box represents the median value. An unpaired two-tailed *t* test was used to calculate significance. Boxplots were generated using Origin Pro 2020b. Image analysis was done using Fiji Ver 1.54f. *B*, representative Total Internal Reflection Fluorescence (TIRF) microscopy images of live CHO cells transiently transfected with BTK^E41K^ or BTK^E41K/R49S^ mCherry in the presence or absence of 1 mM IP_6_ at indicated time points. Scale bar = 10 μm. *C*, the fluorescence intensity at the plasma membrane measured in the transiently transfected live CHO cells with the indicated BTK-mCherry constructs following IP_6_ stimulation is plotted as a function of time. Data are presented as mean values ± SD from three independent experiments. Data analyses were performed using GraphPad Prism version 9.5.1. The t_1/2_ is determined by fitting the decay of the fluorescence intensity to exponential decay. Image analysis was done using Fiji Ver 1.54f. *D*, the plot of fluorescence intensity of BTK^E41K^ mCherry against indicated IP_6_ concentration. Data are presented as mean values ± SD from three independent experiments. Data analyses were performed using GraphPad Prism version 9.5.1. Image analysis was done using Fiji Ver 1.54f.

To study the effect of IP_6_ on the activation profile of BTK, we transiently transfected wild-type and E41K mutant (BTK^E41K^) of BTK-tagged mCherry to the CHO cells and determined the membrane localization (Fig. 4, *A* and *B*). The plasma membrane was marked with Wheat Germ Agglutinin (WGA) fused to Alexa 633 (44). The activation of BTK was determined with a specific anti-pY551 antibody and then imaged by labeling with a FITC-tagged secondary antibody (Fig. 4). The membrane localization of BTK^E41K^ was estimated from the quantification of Pearson’s colocalization coefficient (PCC) of mCherry and Alexa 633 (45). A higher PCC value indicates higher membrane localization. We used a triple mutant (E41K, R28H, and R49S) of BTK as a monomer control (negative control) (Fig. S3, *B*–*D*). We observed that a major fraction of wild-type BTK remains in the cytosol (Fig. 4*A*). In contrast, a significant portion of the BTK^E41K^ localized to the plasma membrane (Fig. 4*B*). Consistent with our *in-vitro* experiments (Figs. 2 and 3), we observed that the mutation at the peripheral lipid binding site (R49S), made in the background of BTK^E41K^, can spontaneously localize to the membrane (Fig. 4*C*). We observed that a minor fraction of BTK^E41K/R49S^ peripheral site mutant is also present in cytosol (Figs. 4*C* and S4*C*). However, the XLA mutation (R28H) impairs the membrane localization of BTK^E41K^ (Fig. 4*D*). As shown previously, we also observed that only the BTK^E41K^ and a minor fraction of BTK^E41K/R49S^ spontaneously phosphorylated Y551 at the plasma membrane (Figs. 4, *B* and *C*, 5*A*, and S3, *B* and *C*), but none of the other two mutants were active (13, 35, 38). These data suggest that the BTK in the CHO cells is mainly recruited to the membrane by binding PIP_3_ to the canonical lipid-binding site of the PH-TH domain (29, 30, 38). The binding of PIP_3_ to the peripheral lipid binding site is required for the transphosphorylation of BTK (22, 30). We asked if BTK^E41K^ is activated only at the plasma membrane or if IP_6_ could stimulate the cytoplasmic fraction of BTK^E41K^.

### IP_6_ increases the cytosolic fraction of active BTK

Next, we focused on how the BTK^E41K^ activation profile at the membrane may be remodeled upon stimulating the transiently transfected CHO cells with IP_6_. Our NMR analysis of CHO cells treated with and without IP_6_ suggests that the IP_6_ spontaneously enters the cell (Fig. S3*E*). We consider that the IP_6_ may enter the cell through pinocytosis (46) and transphosphorylate Y551 of BTK^E41K^ by inducing an IP_6_-dependent dimerization (30). In Figure 4, *A*–*D*, Figs. S3B, and S4, mCherry intensity indicates BTK expression level, FITC indicates the level of Y551 phosphorylation, and WGA-Alexa 633 indicates plasma membrane boundary. We observed that IP_6_ activates the cytosolic fraction of BTK wild-type (Figs. 4*A*, 5*A*, and S4*A*). This is consistent with previous reports (22, 33) that the wild-type BTK predominantly resides in the cytosol, which is activated by IP_6_. In contrast, adding IP_6_ significantly redistributes the membrane-bound fraction of BTK^E41K^ to the cytosol (Figs. 4*B* and S4*B*). The fraction of Y551 phosphorylation for the BTK^E41K^ mutant in the cytosol is now significantly higher than the membrane-bound fraction (Figs. 5*A* and S4*B*). The monomer control stays in the cytosol and cannot be activated by IP_6_ stimulation (Figs. S3, *B*–*D* and S4*E*).

The TIRF imaging of the membrane-bound fraction of BTK^E41K^ shows that the IP_6_ induces rapid redistribution of the kinase from the membrane to the cytosol with a t_1/2_ of 4.2 ± 2.5 min (Fig. 5, *B* and *C*). The BTK^E41K^ mutant demonstrates an ultrasensitivity to IP_6_ concentration. We observed that BTK^E41K^ remains at the membrane till 0.25 mM IP_6_ concentration (Figs. 5*D* and S5). However, at a critical concentration of IP_6_ (0.5 mM), the BTK^E41K^ comes out of the membrane. Our *in-vitro* studies showed that the mutation at the peripheral lipid binding site (R49S) of PH-TH^E41K^ retains the ability to bind IP_6_ to the canonical site (Fig. 3*A* and Table S1). We ask if full-length BTK bearing the same mutation could be activated in cells. We observed that the BTK^E41K/R49S^ mutant remained at the membrane even after being stimulated with IP_6_ and was weakly activated (Figs. 4*C* and S4*C*). Live cell imaging by TIRF microscopy shows that the BTK^E41K/R49S^ mutant has a longer retention time at the plasma membrane, which does not come off the membrane after IP_6_ stimulation throughout the duration of our experiment (Fig. 5, *B* and *C*). Our data indicates that the membrane-localized fraction of BTK^E41K/R49S^ mutant dimerizes by binding PIP_3_ to the intact canonical binding site. We could not detect the Y551 phosphorylation of BTK^E41K/R49S^ mutant in the cytosolic fraction upon IP_6_ stimulation (Figs. 4*C* and 5*A*). Together, this suggests that the binding of IP_6_ to the peripheral site is critical for membrane-independent dimerization of BTK.

**Figure 6:**
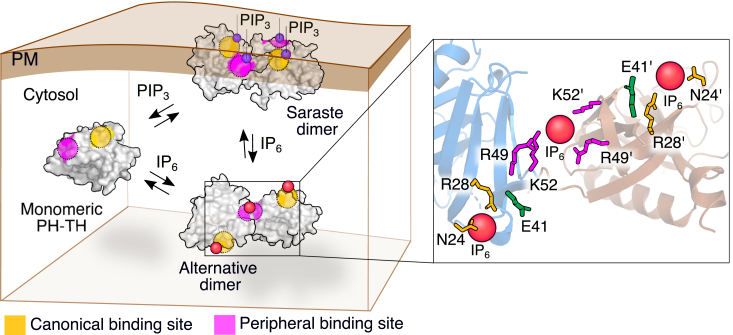
**The proposed model for the IP**_**6**_**-dependent dimerization of the PH-TH domain.** In the absence of stimulation, the PH-TH domain remains in solution as a monomer. The binding of PIP_3_ at the canonical site recruits BTK to the membrane and the Saraste dimer interface is stabilized by PIP_3_ binding to the peripheral site. The canonical and peripheral lipid binding sites are indicated with *yellow* and *magenta*, respectively. IP_6_ promotes an alternate dimer of the PH-TH domain, shifting the equilibrium to a PH-TH dimer in solution. The residues in the canonical and peripheral binding sites are shown in the inset. The IP_6_ molecules are indicated by *red balls*.

Our data suggests that the formation of the PH-TH Saraste dimer is crucial for BTK activation at the membrane or in the cytosol (30) (Fig. 6). However, the inability of IP_6_ to peel the BTK^E41K/R49S^ mutant from the plasma membrane indicates that the IP_6_ may not be competing with the PIP_3_ binding at the canonical lipid binding site. As anticipated, IP_6_ stimulation does not activate BTK^E41K/R28H^ mutated at the canonical binding site mutant (R28H) (Figs. 4*D* and 5*A*), suggesting that the two lipid-binding pockets of BTK are allosterically coupled to the Saraste dimer interface.

## Conclusions

BTK is indispensable for B-lymphocyte development and proliferation, particularly during the maturation of the pre-B-cell stage to the mature stage (7). The PH-TH domain of BTK functions as a master regulator. The activating mutation (E41K) (47, 48) or loss of function mutant (XLA mutation) in the PH-TH domain leads to the manifestation of an immunodeficient phenotype (1, 2). The β3-β4 segment of PH-TH plays an important role in the allosteric crosstalk during the membrane recruitment and dimerization of the kinase (29, 30). One face of the β3-β4 segment constitutes the canonical lipid binding pocket for membrane localization (28). On the opposite side, the β3-β4 segment makes contact with an IP_6_ molecule (22) and laterally interacts with the neighboring PH-TH domain. Therefore, mutation (such as E41K) that stabilizes the β3-β4 segment (lower B-factor) (37) enhances the structural stability of the PH-TH domain (Fig. 2*E*), thereby spontaneously activating the kinase.

Constitutive activation of BTK is often linked to several B-cell malignancies (7). Most BTK inhibitors are competitive inhibitors, competing for the ATP binding pocket. IP_6_ is the only BTK activator reported in the literature. IP_6_-mediated activation of BTK is distinct from IP_4,_ which works synergistically with the PIP_3_ binding for the full activation of Tec family protein tyrosine kinases Itk (49). In contrast, it was speculated that the increased cellular concentration of IP_6_ may outcompete the PIP_3_ and PH-TH binding at the membrane and shift the equilibrium towards the cytosolic fraction (Fig. 4*B*) (22, 50). Counterintuitively, the IP_6_ only perturbs the membrane localization of the BTK^E41K^ but not the peripheral binding site mutant (R49S) (Fig. 4*C*). Together, our data suggests that IP_6_ may not compete with PIP_3_ at the membrane to bind the PH-TH domain.

The crystal structure of the PH-TH domain in the complex with IP_6_ (PDB ID: 4Y94) has four PH-TH domains present in the asymmetric unit (Fig. S6) (22). Two PH-TH domains are dimerized by the lateral interaction between the Saraste dimer interface (Fig. 2*A*) (28, 37). The Saraste dimer is compatible with the simultaneous PIP_3_ binding to the canonical and the peripheral sites at the membrane (29, 30). An alternate homo dimer of PH-TH is also found (Fig. S6) at the asymmetric unit of the PH-TH domain structure (PDB ID: 4Y94). The PH-TH domain dimerizes by an asymmetric interaction between the adjacent β3-β4 segments, forming a pocket. One IP_6_ molecule is sandwiched at the interface of two PH-TH domains, acting as a glue (Fig. 6). This dimer is only observed in the crystal structure of the IP_6_:PH-TH complex and not in the presence of other inositol polyphosphate. Such an IP_6_-dependent PH-TH dimer is incompatible with binding two PIP_3_ molecules simultaneously to the canonical lipid binding site at the membrane while retaining the IP_6_ molecule at the peripheral lipid binding site (Fig. 6). We speculate that IP_6_ may stabilize the alternate dimer over the Saraste dimer. Thus, shifting the equilibrium to the alternate PH-TH dimer, thereby dislodging the BTK^E41K^ from the plasma membrane (Figs. 4*B* and 5*B*). The peripheral site mutant (R49S) cannot form the alternate dimer and remains at the plasma membrane even in the presence of IP_6_ (Fig. 4*C*). The ultrasensitivity of BTK^E41K^ to the IP_6_ concentration (Fig. 5*D*) indicates that the PH-TH functions as a switch that, at a critical IP_6_ concentration, toggles the PH-TH Saraste dimer to the alternate dimer (30).

Our data show that the XLA mutation (R28H) impairs dimerization of the PH-TH domain and IP_6_-dependent phosphorylation of Y551 of BTK (Figs. 4*D* and S3D). The R28H mutation may have impaired response to the T-cell-independent B-cell antigens, explaining why patients with XLA are more susceptible to recurring bacterial and viral infection (1, 2, 3, 4). We speculate that the ability of IP_6_ to activate BTK independent of BCR activation in our experiment opens a new opportunity to develop immune modulators.

## Experimental procedures

### Constructs

The PH-TH domain of BTK wildtype (amino acid residue number 4–170) cloned into pET28-SUMO vector was gifted from Prof John Kuriyan, UC Berkeley. The pMSCV-BTK-mCherry construct was a gift from Hidde Ploegh (Addgene plasmid #50043). The full-length BTK was cloned into the pET28-SUMO vector. For the microscopy studies, the BTK-mCherry was cloned into a pCDNA vector. The point mutations in all the constructs were done by site-directed mutagenesis.

### Expression and purification of BTK PH-TH domain

The PH-TH domain of BTK cloned into pET28-SUMO vector was expressed in *E.coli* BL21-DE3 cells by inducing with 1 mM IPTG and at 18^o^C for overnight (22). The cells were lysed in lysis buffer (25 mM Tris-Cl, 500 mM NaCl, 20 mM Imidazole, 5% Glycerol, 2 mM β-ME, pH 8.5). The protein was purified using a Ni-NTA column, and buffer exchanged to 25 mM Tris-Cl, 400 mM NaCl pH 8. His tag was removed by incubating with ULP1 protease for 12 h, and the PH-TH domain was further purified by gel filtration chromatography. the protein was concentrated and stored at −80^o^C.

### Expression and Purification of full length BTK

The full-length BTK cloned in pET28-SUMO vector was co-transformed with YopH and Trigger factor in *E.coli* BL21-DE3 cells (22). Briefly, the cells were grown in LB supplemented with 50 mg/ml Kanamycin, 35 μg/ml chloramphenicol, and 50 μg/ml streptomycin. The BTK expression was induced with 1 mM IPTG and was grown at 18 ^o^C for 16 h. The cells were lysed in lysis buffer (25 mM Tris-Cl, 500 mM NaCl, 20 mM Imidazole, 5% Glycerol, 2 mM β-ME, pH 8.5) and the protein was purified using Ni-NTA column. His tag was removed by digesting BTK with ULP1 protease for 12 h. The undigested protein was then removed by passing through the second Ni-NTA column and the flow through was concentrated and stored at −80 ^o^C.

### Isothermal titration calorimetry

The isothermal titration calorimetry experiments were performed using MicroCal ITC (GE Healthcare) and MicroCal PEAQ-ITC (Malvern). The PH-TH domain was buffer exchanged to 20 mM HEPES, 150 mM NaCl, pH 7.5. During the titration, 25 μM protein in the cell was titrated with a ligand at the protein-to-ligand ratio of 1: 5.5. All ITC titrations were performed at 20 °C. The initial injection of 0.5 μl was excluded from the data analysis. During the titration, 3 μl of ligand was injected in 13 steps, and each injection step was separated by 180 s. The protein solution was stirred at 300 rpm during the titration.

### Size-exclusion chromatography

Size-exclusion chromatography of various constructs of the PH-TH domain was performed using the Superdex-200 10/300 Gl column. For the apo PH-TH domain, the column was equilibrated with running buffer (25 mM Tris-Cl, 100 mM NaCl, 1 mM DTT, pH 7.2), and 500 μl of 75 μM protein was loaded into the column, at a flow rate of 0.3 ml/min and at 8 °C. The elution profile was recorded, and the retention time of the protein was calculated. For the ligand-bound samples, the protein was mixed with ligand at a protein-to-ligand ratio of 1:4, and incubated at 4 °C for 15 min before loading into the size-exclusion column. Before loading the sample, the column was pre-equilibrated with a running buffer containing the ligand.

### Dynamic light scattering

The Dynamic Light Scattering experiments were conducted using Malvern Zetasizer DLS Detectors using 1.5 ml plastic cuvettes. The samples were prepared in a buffer containing 25 mM Tris-Cl, 100 mM NaCl, 1 mM DTT, and pH 7.2. The experiment included three measurements of ten runs each. The theoretical calculations of the hydrodynamic radius of the proteins were performed using Hydropro (51).

### Thermal denaturation by circular dichroism spectroscopy

The melting temperature of the PH-TH domain was determined from the Circular Dichroism (CD) spectrum recorded at increasing temperatures (20° to 76 °C). For each sample, the CD spectra from 300 to 190 nm were recorded using a Jasco-J1500 spectropolarimeter with a temperature increment of 4 °C. CD spectra were recorded with 5 μM protein in 20 mM Sodium phosphate buffer pH 7.4 and in the absence or in the presence of an equimolar ligand. The measured ellipticity data was converted into molar ellipticity (39).

The ${\Delta G}_{Unfolding}$ was calculated considering the simplest two-step unfolding model, where a protein exists in a folded (F) and an unfolded (U) state as described previously (40, 41). The fraction unfolded [U] protein at a given temperature is:

(1) $$\left[U\right]=\frac{\left(\theta_{T}-\theta_{F}\right)}{\left(\theta_{U}-\theta_{F}\right)}$$

Where, θ_F_ and θ_U_ are the molar ellipticity of the fully folded and unfolded state at 218 nm, θ_T_ is the molar ellipticity at a given temperature. Considering that at a given temperature, the sum of the unfolded and folded [F] fraction of the protein is 1, the equilibrium constant, $K_{Unfolding}$ is:

(2) $$K_{\text{Unfolding}}=\frac{\left[U\right]}{1-\left[U\right]}$$

Thus, free energy of unfolding was derived using ${\Delta G}_{\text{Unfolding}}=-\text{RTln}\left(K_{\text{Unfolding}}\right)$, where R is the universal gas constant (1.98 cal mol^−1^K^−1^). The ΔΔG_unfolding_ was calculated from the difference between the ΔG of the IP_6_ bound ΔG_*holo*_ and ΔG_*apo*_ state, measured at the T_m_ of the corresponding apo PH-TH construct.

### Autophosphorylation assay by immunoblot

The activation of full-length BTK in response to the ligand was determined from the autophosphorylation Y551 or total phosphotyrosine level. Before each reaction, 2 μM BTK was incubated with 100 μM ligand for 10 min. The phosphorylation reaction was performed at 25 °C in a buffer containing 25 mM Tris-Cl (pH 7.5), 2 mM ATP, 10 mM MgCl_2,_ and 1 mM Na_3_VO_4_. The reaction was quenched with SDS–PAGE loading buffer. The autophosphorylation levels of full-length BTK were detected by immunoblot (22). The amount of BTK loaded was determined using an anti-BTK antibody. Densitometric analysis of immunoblots was performed using Fiji software (52).

### Cell-based assay

The IP_6_-mediated activation of BTK was determined by immunofluorescence and immunoblot in Chinese Hamster Ovary (CHO) cell line transiently transfected with BTK-mCherry cloned in pCDNA. The CHO cell line was obtained from the National Center for Cell Science cell repository in Pune, India, and tested for *mycoplasma* contamination. Before activation, transfected cells were serum-starved for 6 h in opti-MEM, followed by incubation with 500 μM IP_6_ for 1 h in opti-MEM. For Immunoblot, cells were washed with ice-cold PBS and then incubated in RIPA buffer [10 mM Tris-Cl (pH 8.0), 140 mM NaCl, 1 mM EDTA, 0.1% SDS, and 1% Triton X-100] containing protease and phosphatase inhibitors (2 mM Benzamidine, 1 mM PMSF and 1 mM Sodium orthovanadate) for 10 min. The cell lysates were sonicated, and protein samples were prepared by heating with 5X loading buffer and resolved in a 6% SDS-PAGE. Protein samples were transferred onto the PVDF membrane at 15 V for 1 h, followed by blocking with 5% skimmed milk in 1X TBS containing 0.1% TWEEN-20 for 1 h at room temperature. After blocking, the blot was incubated overnight at 4 °C with the primary antibody (1:1000) diluted in 3% skimmed milk. After incubation, the blot was washed three times with 1X TBST (0.1% TWEEN-20), followed by incubation with a secondary antibody (1:2000) diluted in 3% skimmed milk. Blots were washed three times with 1X TBST and developed using the Clarity Western ECL substrate kit (Bio-Rad). Densitometry analysis of immunoblots was performed using Fiji Ver 1.54f (52).

For the immunofluorescence study, Cells were washed with PBS once, followed by incubation with WGA (20 μg/ml) in PBS at 37 °C for 1 min. After incubation, cells were washed with PBS and immediately fixed with 4% paraformaldehyde in PBS for 30 min at room temperature. After fixation, cells were washed five times with 1x PBS and permeabilized with 0.2% PBST for 5 min at room temperature. Cells were blocked with 1% BSA for 1 h at room temperature, followed by staining with anti-551pY primary antibody (1:100 dilution) in blocking buffer overnight at 4 °C. After incubation, cells were incubated with a FITC-conjugated secondary antibody in a blocking buffer for 2 h at room temperature. Finally, coverslips were mounted with prolonged gold. Coverslips were washed three times with 1X PBS in between each step. Details of the antibody used are summarized in Table S3.

### Confocal microscopy and image analysis

All images were acquired with the Leica SP8 confocal platform using an oil immersion HC PL APO CS2 63 × objective (NA 1.4) at 3.0X digital zoom. Image scanning was done in bidirectional mode at 500 Hz. The level of BTK on the membrane or cytoplasm was determined from the intensity of mCherry measured at λ_ex_ = 552 nm and λ_em_ = 576 to 651 nm, respectively. The Y551 phosphorylation was determined from the intensity of FITC tagged to the secondary antibody and measured at λ_ex_ = 488 nm and λ_em_ = 505 to 531 nm. The plasma membrane was stained with Wheat Germ Agglutinin (WGA) fused to Alexa 633 and imaged at λ_ex_ = 633 nm, λ_em_ = 647 to 692 nm, respectively. ROIs were drawn manually using the Fiji freehand tool (Ver 1.54f) (52). ROI1 marked the outer boundary of the cell perimeter labeled with WGA, ROI2 marked the inner boundary of the cell perimeter, and ROI3 marked the nucleus. The ROI1 provides the total cell intensity. The intensity of mCherry or FITC at the membrane was determined by subtracting the respective intensities of ROI2 from ROI1. Whereas cytoplasmic intensity of mCherry or FITC was determined by subtracting the respective intensities of ROI3 from ROI2. The membrane localization of the BTK-mCherry construct was determined from the quantification of Pearson’s colocalization coefficient (PCC) (45).

### Total internal reflection fluorescence (TIRF) microscopy

For Total internal reflection fluorescence (TIRF) microscopy, CHO cells were seeded in a glass bottom dish and transfected with 500 ng DNA. After 16 h of post-transfection, cells were serum starved by incubating in Opti-MEM serum-free media for 6 h. The images of lived CHO cells were recorded for 10 min before and after IP_6_ treatment. The TIRF images were acquired on a Nikon Eclipse Ti2 inverted microscope equipped with a Nikon 100 × 1.49 numerical aperture oil-immersion TIRF objective, a TIRF illuminator, a Perfect Focus system (PFS), and a motorized stage. The critical angle and the refractive index were set to 63° and 1.515, respectively (53). The samples were excited at 561 nm using an LU-N4 laser unit (Nikon, Tokyo, Japan) with solid-state lasers. Image acquisition was done using the Nikon NIS-Elements software. Live cell images were acquired at 1-min intervals for 10 min using an EMCCD camera, with an exposure time of 200 ms.

The images were processed and analyzed using Fiji. Out-of-focus fluorescence in the acquired images is removed by deconvolution (54). Image ROIs were drawn using the elliptical tool for each cell. The fluorescence intensity of each frame was quantified using “create spectrum jru V1” routine of Fiji. The half-life of fluorescence intensity at the plasma membrane was determined by fitting the fluorescence decay at indicated time points to a first-order kinetic equation using GraphPad Prism version 9.5.1.

### Analysis of intracellular IP_6_ by NMR spectroscopy

The intracellular IP_6_ was extracted with TiO_2_ (55) and analyzed by NMR spectroscopy (56). Briefly, CHO cells were grown to a confluency of 2.6 × 10^7^ cells at 37 °C and 5% CO_2_. Before IP_6_ treatment, cells were serum starved for 6 h, and then 5 mM IP_6_ in 25 mM Tris buffer (pH 7.4) was added and incubated for 30 min. The cells were then washed twice with ice-cold PBS to remove excess IP_6_ and lysed by incubating with ice-cold 1M Perchloric acid for 10 min. The cell extract was centrifuged at 14,000*g* for 10 min to remove the cell debris. The TiO_2_ slurry was prepared by washing 200 mg of TiO_2_ powder with 5 ml of deionized water and then resuspended in 1M Perchloric acid. The cell extracted in perchloric acid was added to the TiO_2_ and incubated at 4 °C for 15 min. The supernatant was removed by centrifugation, and the bound phosphate compounds were eluted from TiO_2_ with 2.8% NH_4_OH. The eluate was lyophilized overnight and resuspended in a Sodium Phosphate buffer (pH 6.5) containing 50 mM KCl. The sample was again lyophilized to remove any residual water and resuspended in 100% D_2_O for the NMR analysis. Commercially purchased pure IP_6_ was used as a standard. The 1D proton NMR spectrum was recorded at a ^1^H frequency of 500 MHz. The relaxation delay was set at 5 s and the spectral width of 12,500 Hz. The data was analyzed with Bruker Topspin 4.4.0.

## Data availability

All the relevant data are contained within this article and in the supporting information.

## Supporting information

This article contains supporting information.

## Conflict of interest

The authors declare that they have no conflict of interest with the contents of this article.
