# Supplementary material for: E41K mutation activates Bruton’s tyrosine kinase by stabilizing an inositol hexakisphosphate-dependent invisible dimer
Source: J Biol Chem. 2024 Jul 4;300(8):107535. doi: 10.1016/j.jbc.2024.107535 (PMC11338949; doi:10.1016/j.jbc.2024.107535)
Supplement: Supporting Information [file mmc1.pdf]

SUPPORTING INFORMATION

E41K Mutation Activates Bruton's Tyrosine Kinase by Stabilizing an Inositol Hexakisphosphate dependent Invisible Dimer

Subhankar Chowdhury[a]+, Manas Pratim Chakraborty[a]+, Swarnendu Roy[a]+ , Bipra Prasad Dey[a], Kaustav Gangopadhyay[a] and Rahul Das[a],[b]*

[a] Department of Biological Sciences, Indian Institute of Science Education and Research Kolkata, Mohanpur campus, Mohanpur-741246, India

[b] Centre for Advanced Functional Materials, Indian Institute of Science Education and Research Kolkata, Mohanpur campus, Mohanpur-741246, India

[+] These authors made equal contributions.

Manuscript

Corresponding authors

Rahul Das: rahul.das@iiserkol.ac.in

Keywords: Kinase, Cell signaling, BTK, Inositol Hexakisphosphate, and B-cell receptor

Table S1: Thermodynamic parameters for binding of IP_6 to the BTK PH-TH variants derived from Isothermal Titration Calorimetry

PH-TH Constructs	K_{d1} (nM)	ΔH_1 (cal/mole)	K_{d2} (nM)	ΔH_2 (cal/mole)
E41K	152 ± 18	-4749 ± 159	1275 ± 366	-5098 ± 2360
E41K/R28H	2421 ± 151	-1562 ± 1008	-	-
E41K/R49S	622 ± 77	-4160 ± 550	-	-
E41K/R28H/R49S	-	-	-	-
R28H/R49S	-	-	-	-

Table S2: $\Delta G_{unfolding}$ and $\Delta\Delta G_{unfolding}$ of Apo and IP_6 bound PH-TH domain of BTK

**PH-TH Construct**	**T_m(°C)**	**$\Delta G_{Unfolding}$** ** **(kcal/mol)**	**$\Delta\Delta G_{Unfolding}$ (kcal/mol)***
WT Apo	55.82±0.92	0.001±0.018	-
WT + IP_6	58.87±1.17	0.742±0.205	0.741±0.223
E41K Apo	54.53±0.65	0.012±0.228	-
E41K + IP_6	60.03+0.76	1.496±0.557	1.485±0.785
E41K + IP_3	54.81±0.21	0.056±0.06	0.044±0.288
E41K/R28H/R49S Apo	44.69±0.51	0.037±0.146	-
E41K/R28H/R49S+IP_6	46.71±0.11	0.524±0.029	0.487±0.176

** The $\Delta G_{unfolding}$ was calculated at the Tm of the *apo* state of the respective PH-TH domain

* $\Delta\Delta G_{Unfolding} = \Delta G_{holo} - \Delta G_{apo}$

Table S3: Details of antibodies

Antibodies	Source	Details
BTK (D3H5) Rabbit mAb	Cell Signaling Technology, Danvers, MA, USA	Cat # 8547S Lot: 13
Recombinant Anti-BTK (phospho Y551) antibody [EP267Y]	Abcam (Waltham, MA 02453, USA)	Cat # ab40770 GR111548-5
Recombinant Anti-Phosphotyrosine antibody [EPR16871]	Abcam (Waltham, MA 02453, USA)	Cat # ab179530 GR198792-25
Rabbit HRP Secondary antibody	Abcam (Waltham, MA 02453, USA)	Cat # 50095 Lot: 2960660
GAPDH rabbit polyclonal antibody	BioBharati Life Science Pvt Ltd	Cat # BB-AB0060 Lot: 011501
Rabbit FITC-conjugated secondary antibody	Abcam (Waltham, MA 02453, USA)	Cat # Ab6885 Lot: GR3391568-I

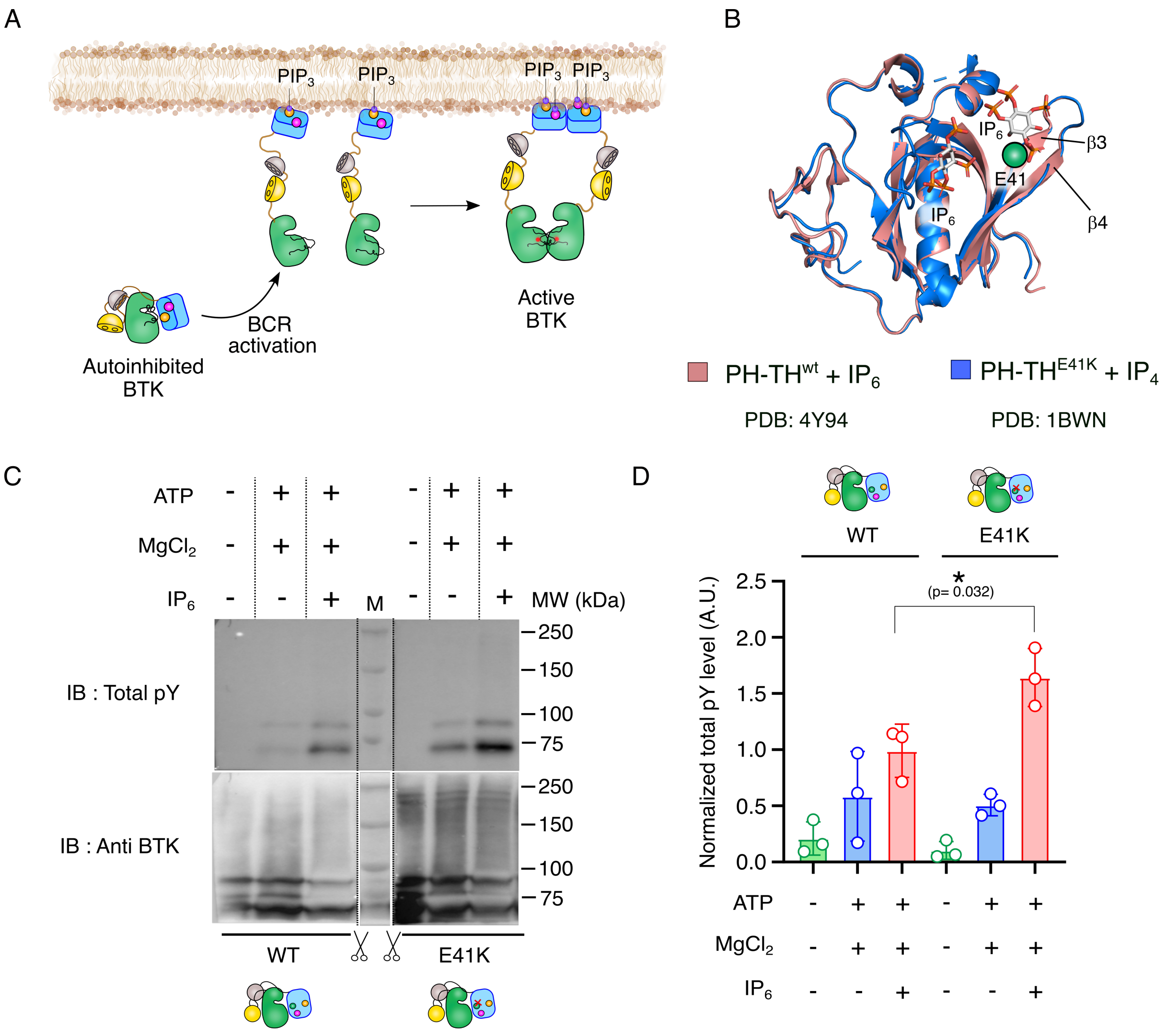

Figure S1. BCR-mediated activation of BTK

(A) Schematic representation of PIP_3 mediated membrane recruitment of BTK upon BCR activation.

(B) Structure alignment of PH-TH^{WT} in complex with IP_6 (PDB ID: 4Y94) (1) and PH-TH^{E41K} domain in complex with IP_4 (PDB ID: 1BWN) (2).

(C) Rrepresentative immunoblot showing the IP_6-dependent activation of purified full-length wild-type (WT) BTK and BTK^{E41K} mutant. In the top panel, the level of autophosphorylation is determined with a total anti-phosphotyrosine antibody, and the bottom panel shows the loading control.

(D) Densitometric analysis of immunoblots of the IP_6-dependent activation of BTK shown in panel (C). Data are presented as mean values ± SD from three independent experiments. Data analyses were performed using GraphPad Prism version 9.5.1. An unpaired two-tailed t-test was used to calculate significance.

A

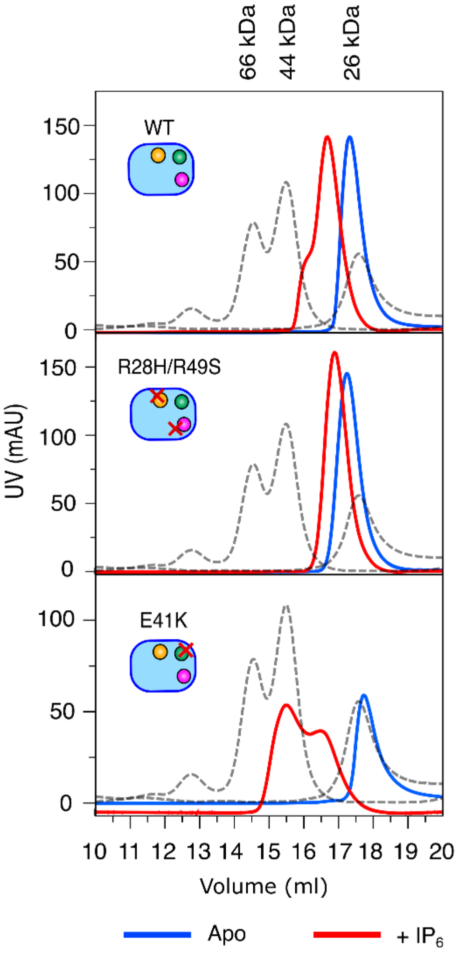

B

Oligomeric state of PH-TH	Molecular Weight (kD)	Expected Hydrodynamic radius(nm)
Monomer	19	2.20
Dimer	38	3.00
Tetramer	76	3.76

C

PH-TH	Hydrodynamic radius(nm) - IP_6	Hydrodynamic radius(nm) + IP_6
WT	2.42 ± 0.46	2.18 ± 0.10
E41K	2.57 ± 0.67	3.14 ± 0.48
R28H/R49S	2.11 ± 0.30	2.20 ± 0.19
E41K/R28H/R49S	2.55 ± 0.21	2.67 ± 0.27

D

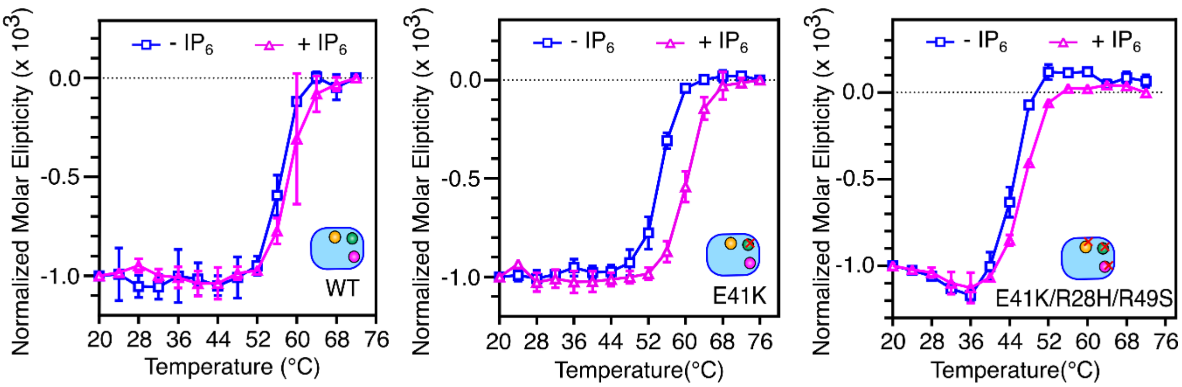

Figure S2. Characterization of the IP_6-dependent PH-TH dimer in solution

A) Representative gel-filtration elution profiles of indicated PH-TH constructs measured in the presence or absence of IP_6. The dotted line represents the elution profile of a standard protein mixture comprised of BSA (66 kDa), Ovalbumin (44 kDa), and ULP1 (26 kDa). The plots were generated by XMGRACE Ver 5.1.25.

B) Predicted Hydrodynamic radius of different oligomeric states of PH-TH domain of BTK calculations using Hydropro software (3).

(C) The hydrodynamic radius of BTK PH-TH domain variants in the presence or absence of IP_6 was measured using Dynamic Light Scattering (DLS).

(D) Thermal denaturation profiles of indicated PH-TH constructs in the presence (magenta) or absence (cyan) of IP_6. Data are presented as mean values ± SD from three independent experiments. The solid line represents the fitting to the Boltzmann Sigmoidal equation using GraphPad Prism version 9.5.1(GraphPad Software, LLC.). Data analyses were performed using GraphPad Prism version 9.5.1.

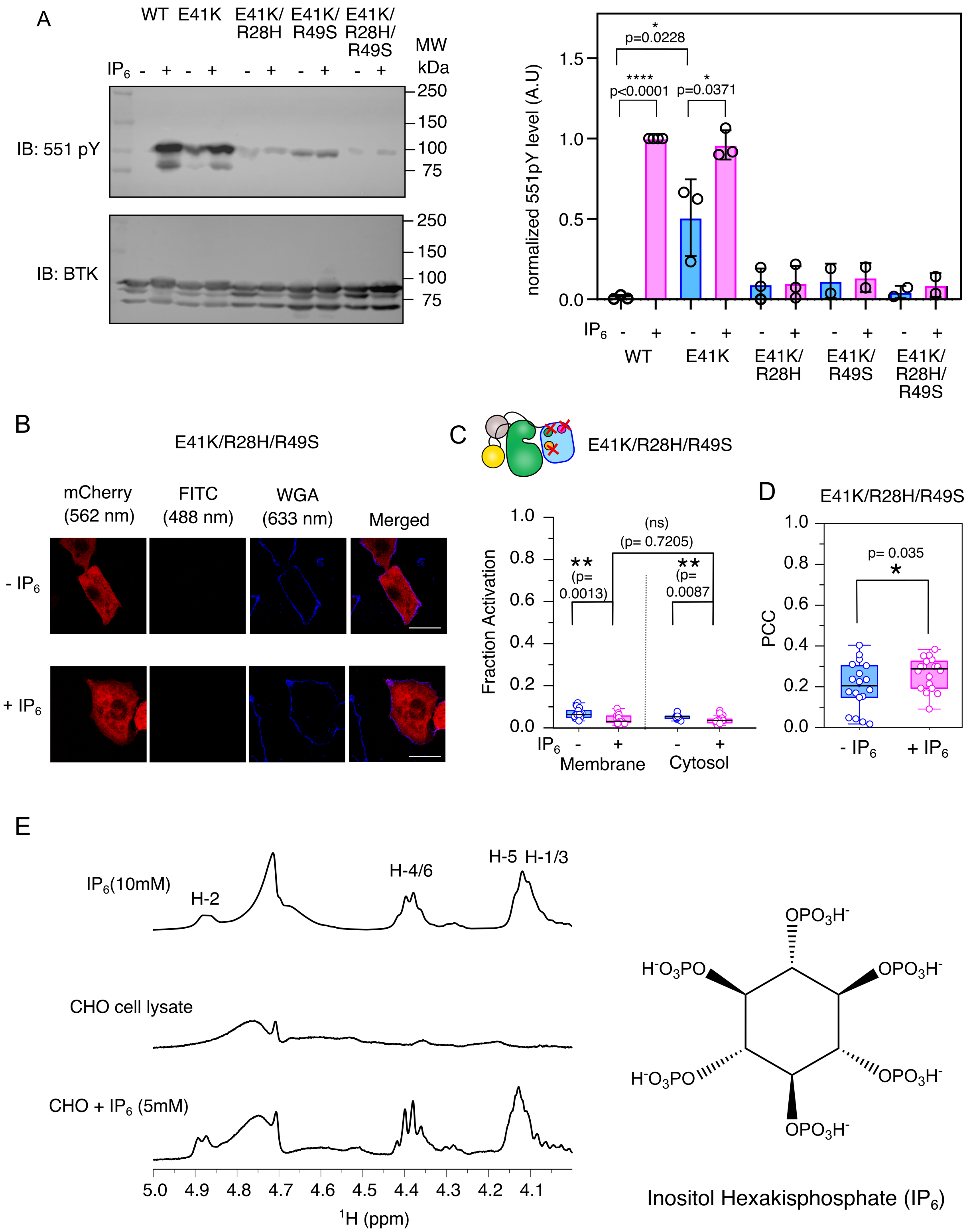

Figure S3. IP_6-mediated activation of BTK in CHO cells

(A) The left panel is the representative immunoblot of Y551 phosphorylation level in the indicated construct of BTK transiently expressed in the CHO cell line in the presence and absence of IP_6. The densitometric analysis of the immunoblot is on the right. Data are presented as mean values ± SD from five independent experiments. Data analyses were performed using GraphPad Prism version 9.5.1.

B) Confocal images of BTK E41K/R28H/R49S mutant in the presence (bottom) or absence (up) of IP_6. The BTK expression level is shown in red (λ_{ex}= 552 nm, λ_{em}= 586-651 nm), and the phosphorylation status is shown in green (λ_{ex}= 488 nm, λ_{em}= 505-531 nm). The blue represents the plasma membrane stained with Wheat Germ Agglutinin (WGA) fused to Alexa 633 (λ_{ex}= 633 nm, λ_{em}= 647-692 nm). Scale bar = 30 µm.

C) Quantification of colocalization of BTK E41K/R28H/R49S mutant and WGA by Pearson's correlation coefficient. n= 22-25 over three independent experiments. Boxplots represent quartiles. The data points outside the whisker range are set as outliers. The black line inside the box represents the median value. boxplots were generated using Origin Pro 2020b. Image analysis was done using Fiji Ver 1.54f (4).

D) The plot of fraction phosphorylated for the BTK E41K/R28H/R49S mutant transiently expressed in CHO cell lines. n= 22-25 over five independent experiments. Boxplots represent quartiles. The data points outside the whisker range are set as outliers. The black line inside the box represents the median value. boxplots were generated using Origin Pro 2020b. An unpaired two-tailed t-test was used to calculate significance.

E) One-dimensional ^{1}H NMR spectra of IP_6 (top) and IP_6 extracted from CHO cell line in untreated (middle) and treated (bottom) with IP_6. The chemical structure of IP_6 is presented on the right.

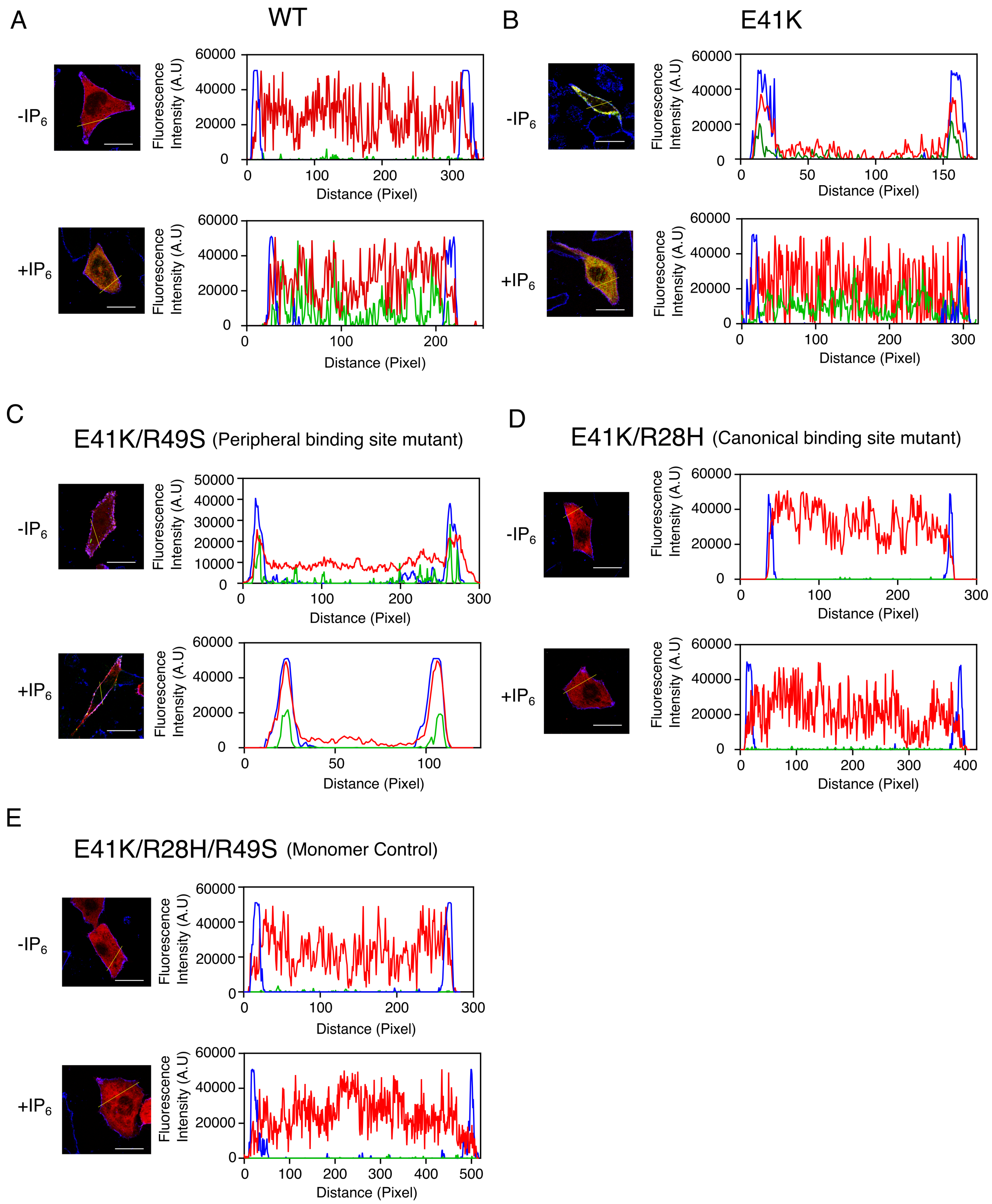

Figure S4. (A-E) Intensity plots of indicated BTK constructs transiently expressed in CHO cell line, in the presence or absence of IP_6. Figures S4 A-D are reused from Figure 4, and Figure S4E is reused from Figure S3B. The BTK expression level is shown in red, and the phosphorylation level of Y551 is shown in green. The blue represents the plasma membrane stained with Wheat Germ Agglutinin (WGA) fused to Alexa 633. Image analysis was done using Fiji Ver 1.54f. The plots were generated using GraphPad Prism version 9.5.1. Scale bar = 30 μm.

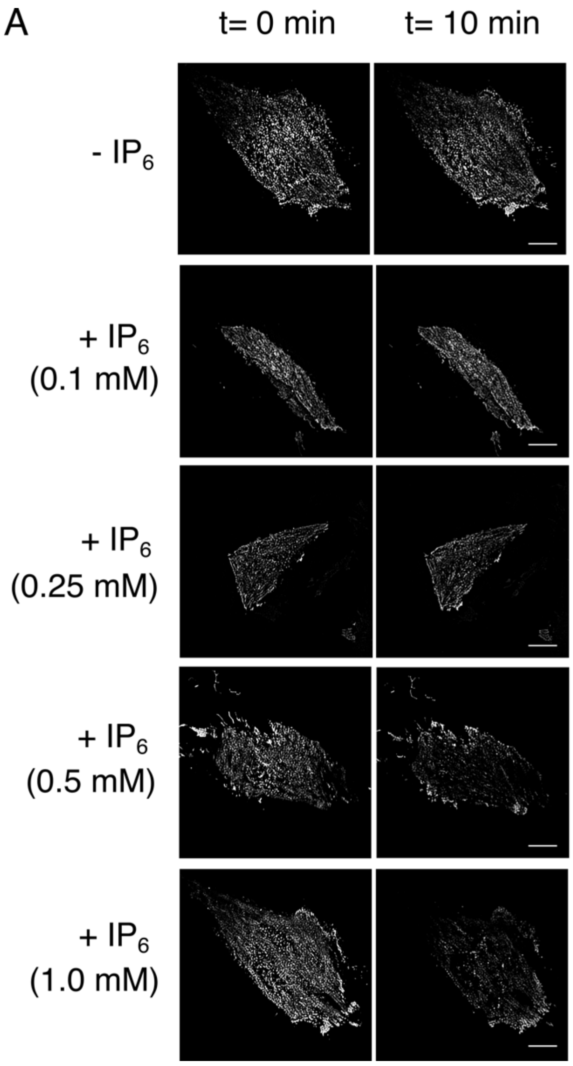

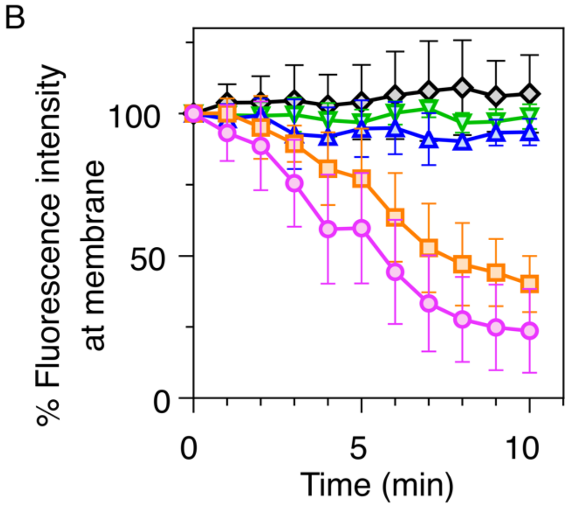

IP_6 (mM)	0	0.1	0.25	0.5	1.0
$t_{1/2}$ (min)	ND	ND	ND	6.4 ± 2.6	4.2 ± 2.5

Figure S5. Measurement of resident time of BTK^{E41K} mutant at the plasma membrane.

(A) Representative total internal reflection fluorescence (TIRF) microscopy images of live CHO cell line transiently transfected with mCherry tagged BTK^{E41K} before (t = 0 min) and after (t = 10 min) treating with the indicated concentration of IP_6. Scale bar = 10 µm.

(B) Plot of membrane fluorescence intensity of BTK^{E41K}-mCherry versus time following IP_6 treatment measured in the transiently transfected CHO cells. The table below shows the half-life of resident time ($t_{1/2}$) of BTK^{E41K} mutant at the plasma membrane, measured at the indicated IP_6 concentration. Data are presented as mean values ± SD from three independent experiments. Data analyses were performed using GraphPad Prism version 9.5.1. The $t_{1/2}$ is determined by fitting the decay of the fluorescence intensity to exponential decay. Image analysis was done using Fiji Ver 1.54f.

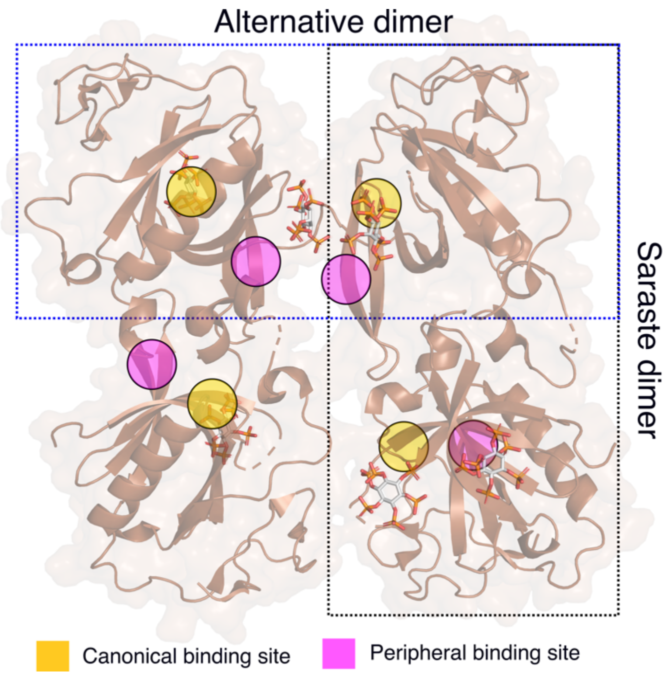

Figure S6. Cartoon representation of PH-TH molecule in the asymmetric unit of the PH-TH: IP_6 crystal structure (PDB: 4Y94) (1). The Saraste dimer and the alternative dimers are shown in the box.

Supplementary References

1. Wang, Q., Vogan, E. M., Nocka, L. M., Rosen, C. E., Zorn, J. A., Harrison, S. C., and Kuriyan, J. (2015) Autoinhibition of Bruton's tyrosine kinase (Btk) and activation by soluble inositol hexakisphosphate. *Elife* **4**, e06074
2. Baraldi, E., Carugo, K. D., Hyvönen, M., Surdo, P. L., Riley, A. M., Potter, B. V., O'Brien, R., Ladbury, J. E., and Saraste, M. (1999) Structure of the PH domain from Bruton's tyrosine kinase in complex with inositol 1, 3, 4, 5-tetrakisphosphate. *Structure* **7**, 449-460
3. Ortega, A., Amorós, D., and De La Torre, J. G. (2011) Prediction of hydrodynamic and other solution properties of rigid proteins from atomic-and residue-level models. *Biophysical journal* **101**, 892-898
4. Schindelin, J., Arganda-Carreras, I., Frise, E., Kaynig, V., Longair, M., Pietzsch, T., Preibisch, S., Rueden, C., Saalfeld, S., Schmid, B., Tinevez, J. Y., White, D. J., Hartenstein, V., Eliceiri, K., Tomancak, P., and Cardona, A. (2012) Fiji: an open-source platform for biological-image analysis. *Nat Methods* **9**, 676-682
